# Reappraisal of the envenoming capacity of *Euchambersia mirabilis* (Therapsida, Therocephalia) using μCT-scanning techniques

**DOI:** 10.1371/journal.pone.0172047

**Published:** 2017-02-10

**Authors:** Julien Benoit, Luke A. Norton, Paul R. Manger, Bruce S. Rubidge

**Affiliations:** 1 Evolutionary Studies Institute, University of the Witwatersrand, Johannesburg, Gauteng, South Africa; 2 School of Anatomical Sciences, University of the Witwatersrand, Johannesburg, Gauteng, South Africa; 3 School of Geosciences, University of the Witwatersrand, Johannesburg, Gauteng, South Africa; College of the Holy Cross, UNITED STATES

## Abstract

*Euchambersia mirabilis* is an iconic species of Permo-Triassic therapsid because of its unusually large external maxillary fossa linked through a sulcus to a ridged canine. This anatomy led to the commonly accepted conclusion that the large fossa accommodated a venom gland. However, this hypothesis remains untested so far. Here, we conducted a μCT scan assisted reappraisal of the envenoming capacity of *Euchambersia*, with a special focus on the anatomy of the maxillary fossa and canines. This study shows that the fossa, presumably for the venom-producing gland, is directly linked to the maxillary canal, which carries the trigeminal nerve (responsible for the sensitivity of the face). The peculiar anatomy of the maxillary canal suggests important reorganisation in the somatosensory system and that a ganglion could possibly have been present in the maxillary fossa instead of a venom gland. Nevertheless, the venom gland hypothesis is still preferred since we describe, for the first time, the complete crown morphology of the incisiform teeth of *Euchambersia*, which strongly suggests that the complete dentition was ridged. Therefore *Euchambersia* manifests evidence of all characteristics of venomous animals: a venom gland (in the maxillary fossa), a mechanism to deliver the venom (the maxillary canal and/or the sulcus located ventrally to the fossa); and an apparatus with which to inflict a wound for venom delivery (the ridged dentition).

## Introduction

Among Synapsida (which includes extant mammals and all species more closely related to them than to sauropsids), non-mammalian Therapsida are an extinct, but once very successful, radiation of basal synapsids. Their diversity included rat to rhinoceros sized herbivorous species, large carnivores and small insectivores, which reflect their ecological domination over terrestrial vertebrate biota throughout the Middle Permian and Early Triassic (-272 to -237Ma) [1, 2]. *Euchambersia mirabilis* Broom, 1931 (Therocephalia, Akidnognathidae) from the Late Permian *Cistecephalus* Assemblage Zone (~257Ma) of the Beaufort Group of the Karoo Supergroup (South Africa), is possibly one of the most fascinating and enigmatic representatives of this early radiation of mammalian forerunners [1, 3]. Because *Euchambersia* displays a huge and deep maxillary fossa associated with a ridged canine, it is considered to be the most promising case of a venomous non-mammalian therapsid [1, 2, 4, 5]. This implies that a hypothesized specialized gland (situated inside the maxillary fossa) was capable of producing a secretion that was delivered into a target animal *via* a ridged canine [6]. If this is true, *Euchambersia* represents the earliest known venomous terrestrial vertebrate, and one of the best supported cases of an extinct venomous species. This makes *Euchambersia* a reference taxon for authors who wish to address the presence of a venomous bite in other extinct taxa, as in other species of non-mammalian therapsids, but also in archosauromorphs, dinosaurs, mammals, and even conodonts ([1, 2, 5, 7–21], see ref [22] for a review).

Currently, previously unknown venomous capabilities are continuously being recognised amongst extant mammals. Toxin producing glands have evolved independently in mammals in at least four orders; Eulipotyphla, Monotremata, Chiroptera and possibly Primates [23]. Coincidently, it has also been proposed that mammals could have been primitively venomous [24, 25], and thus from a palaeobiological perspective, *Euchambersia* is important in providing physiological insight into the deep roots of mammalian evolution [17].

Consequently, the reliability of the hypothesis of the envenoming capacities of the bite in *Euchambersia* not only affects perceptions of the early radiation and the diversity of mammalian and non-mammalian therapsids, but also strongly impacts on palaeobiological reconstructions of a wide variety of extinct vertebrates, and could even influence understanding of the origin and evolution of venom in vertebrates as a whole. Accordingly, this hypothesis should be based on rigorous reasoning.

The venomous *Euchambersia* hypothesis has never been seriously questioned since it was proposed by Nopcsa in 1933 [4], rather it has been unanimously accepted without rigorous testing (e.g. [5, 7, 9]), though a few authors have expressed reservations (e.g. [26]). The recent resurgence of interest in the venomous *Euchambersia* hypothesis is based mainly on the mistaken assumption that the canine of *Euchambersia* is deeply grooved [22] which makes it reminiscent of the opisthoglyphous maxillary fangs of some colubrid, elapid and atractaspid snakes or a *Solenodon* incisor [1, 2, 11, 13, 16–21, 27, 28]. A review of the literature [22] shows that the original descriptions of *Euchambersia* by Broom [29, 30] and Mendrez [26] respectively state that the canine has a “very prominent ridge” and is “canelée” (which translates into “costulated” or “ribbed”), which clearly indicates a ridge on the canine, not a groove. The depiction of a grooved canine only appeared later, in works published since 1986 (e.g. [11, 13, 27]). In addition, it has also been demonstrated that the presence of a longitudinal canine groove, or ridge, would not, by itself, be sufficient to infer a venomous bite in extinct species as a wide range of non-venomous mammalian (e.g. coati, hippopotamus, bats, and baboons), and non-mammalian (e.g. Nile crocodile, many snakes) species exhibit ridged or grooved tooth morphologies [16, 28, 31, 32]. These issues challenge the possibility that *Euchambersia* was venomous, and force a reappraisal of this hypothesis. If it proves false, then many historical palaeobiological interpretations favouring venomous extinct species based on the *Euchambersia* model would need to be revised, especially amongst therapsids and mammals such as *Megawhaitsia* and *Ichibengops* (e.g. [13, 16, 17]), and also in a variety of extinct archosaurs, including dinosaurs (e.g. [11, 12, 14, 19, 20]), and even in a conodont [18].

Despite its relevance for the venom gland hypothesis, no comprehensive description of the dentition of *Euchambersia mirabilis* has yet been undertaken as neither of the two known specimens preserves the full set of anterior teeth. Additionally, the preserved teeth all have damaged crowns. Thus, without the use of destructive sampling techniques, it has not been previously possible to conduct such a study on the preserved dentition. With the advent of X-ray microtomography, non-destructive surveys can be conducted on fossilised skulls without damage to these rare, valuable and non-renewable heritage objects. Over the last decade, scanning technology has spread widely into palaeontology laboratories for the study of important fossils (e.g. [33–35]). A reappraisal of the external and internal anatomy of the rostrum of both skulls of *Euchambersia* using micro X-ray computed tomography (μCT) brings new understanding of the function(s) of the mysterious maxillary fossa and the ridged dentition. Here we redescribe the morphology of the teeth and the structures associated with the maxillary fossa to shed new light on the hypothesized envenoming capacities of *Euchambersia*.

## Material and methods

**Fig 1:**
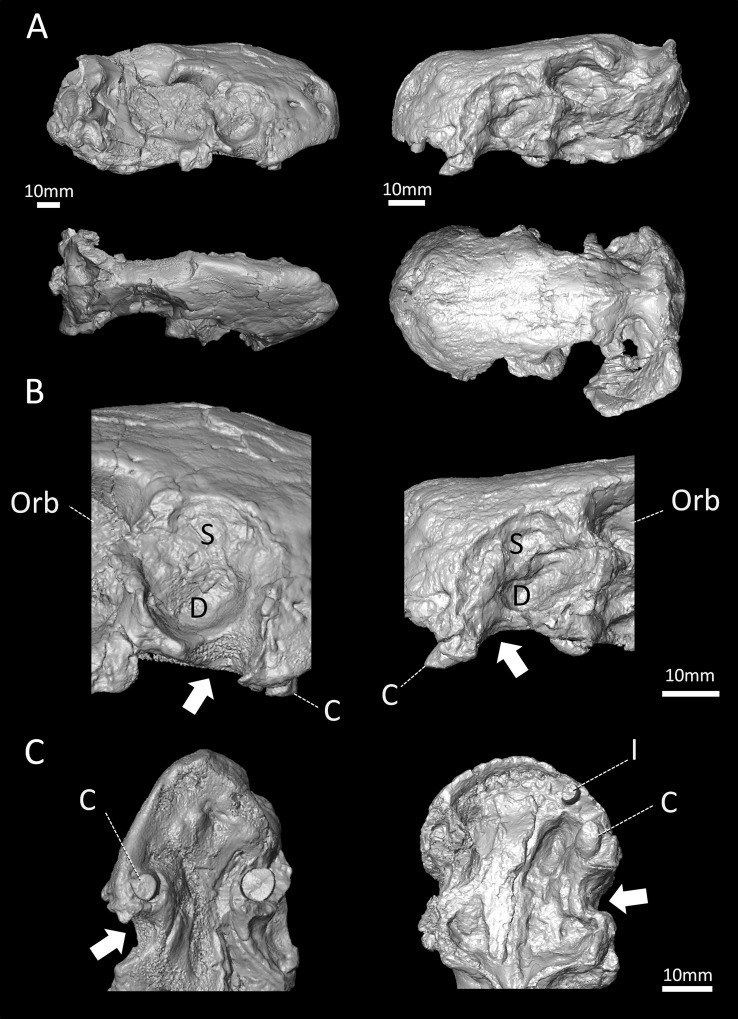
Three-dimensional rendering of the skulls of *Euchambersia mirabilis*. A, NHMUK 5696 (right) and BP/1/4009 (left). B, C close-up views of their respective maxillary fossa in lateral (B) and ventral (C) views showing the sulcus leading from the fossa to the buccal cavity (white arrows). Abbreviations: D, deep part of the maxillary fossa; C, canine; I, incisor; Orb, orbit; S, shallow part of the maxillary fossa.

**Fig 2:**
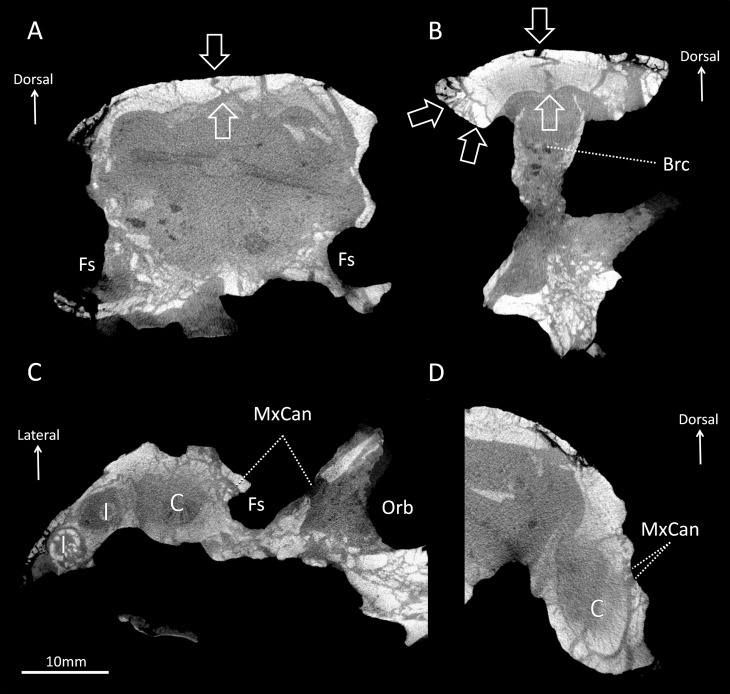
Micro-CT sections through BP/1/4009. A, cross section through the nasal cavity at the level of the fossae; B, cross section through the braincase at the level of the orbits; C, axial section through the snout; D, cross section through the snout at the level of the right canine.

Specimens: Only two specimens of *Euchambersia mirabilis* are known. The holotype, NHMUK R5696 (Fig 1A left) is a large and distorted skull, discovered at the beginning of the 20th century [29]. The referred specimen, BP/1/4009 (Fig 1A right) is a smaller, undistorted, but poorly preserved skull discovered in 1966 [3]. Both come from the same horizon within the *Cistecephalus* Assemblage Zone [3] and have a similar large maxillary fossa which, amongst other characters supports their assignation to the same species (Fig 1) [3, 27]. No lower jaw is known. Specimen BP/1/4009 is likely a younger individual than NHMUK R5696 because of its smaller skull size (Table 1) and the incomplete fusion of its cranial sutures (Fig 2A and 2B).

**Table 1:** Measurements.

	Basal skull length (mm)	Height of the right fossa (mm)	Width of the right fossa (mm)	Height of the left fossa (mm)	Width of the left fossa (mm)	Average diameter of the fossae	Sagittal snout length from interorbital (mm)	Fossa/ snout ratio
NHMUK 5696 (original)	122	30	32	31	25	30	69	43%
NHMUK 5696 (retrodeformed)	116	24	27	29	33	28	58	49%
BP/1/4009	80	20	15	20	26	20	53	38%

Table legend. Snout length was measured from the middle of the orbits. Fossa/ snout ratio is the quotient between the average diameter of the fossae and snout length.

Micro-computed tomography: For this study, *Euchambersia mirabilis* was compared to another akidnognathid therocephalian, *Olivierosuchus parringtoni* (BP/1/3849), to the baurioid therocephalian *Bauria cynops* (BP/1/1180), to the basal cynodont *Thrinaxodon liorhinus* (BP/1/5558) [36], the natural mummy of a venomous snake *Hemachatus haemachatus* (MS-no catalogue number) and the dry skull of a non-venomous snake *Python* sp. (MS-NM46).

Specimens of *Euchambersia* (BP/1/4009), *Olivierosuchus* (BP/1/3849), *Bauria* (BP/1/1180), *Python* (MS-NM46) and *Hemachatus* were scanned separately in the Microfocus X-ray CT facility at the Evolutionary Studies Institute (ESI) of the University of the Witwatersrand using a *Nikon Metrology XTH 225/320 LC* dual-source industrial CT system (see www.wits.ac.za/microCT for additional information on the system). The voxel size is 0.050mm (110kV, 150μA) for *Euchambersia*, 0.0655mm (135kV, 200μA) for *Olivierosuchus*, 0.0668mm (140kV, 240μA) for *Bauria*, 0.0556mm for *Python* and 0.0286mm (80kV, 80μA) for *Hemachatus*. These CT data used for this work are curated on a server at the microfocus CT scanning laboratory of the Evolutionary Studies Institute. Requests concerning access to μCT scan data should be sent to the Wits CT facility manager, K. Jakata: Kudakwashe.Jakata@wits.ac.za.

The holotype of *Euchambersia*, NHMUK R5696, was scanned at the NHMUK (London, United-Kingdom) using a *Metris X-Tek HMX ST 225* CT scanner with a voxel size of 0.066mm (215kV, 170μA). Data of this specimen are curated at the Core Research Laboratories of NHMUK. For enquiries, contact the head of imaging F. Ahmed: f.ahmed@nhm.ac.uk. The ESI specimen of *Thrinaxodon* (BP/1/5558) was scanned at the European Synchrotron Radiation Facility (ESRF) with a voxel size of 0.035mm (see [36] for additional details). Origina data of this specimen are curated at the ESRF. For enquiries, contact the head of imaging V. Fernandez: vincent.fernandez@esrf.fr.

Virtual Reconstruction: Three dimensional rendering and segmentation of data pertaining to the dentition was performed using VG Studio MAX 2.2.5 (Volume Graphics, Heidelberg, Germany). For both specimens of *Euchambersia*, teeth were segmented as separate structures from the surrounding bone and matrix. Segmentation of the teeth of BP/1/4009 was undertaken using the semiautomatic 3-D ‘region growing’ tool. Due to lower contrast between tooth and bone in the scan of NHMUK R5696, this tool could not be used, and segmentation of the teeth was completed manually using the ‘polygon lasso’ and ‘polyline’ tools. Three-dimensional renderings of the internal structure of the maxillary canal and other structures related to the maxillary fossa were obtained using manual segmentation under Avizo 8 (FEI VSG, Hillsboro OR, USA).

**Fig 3:**
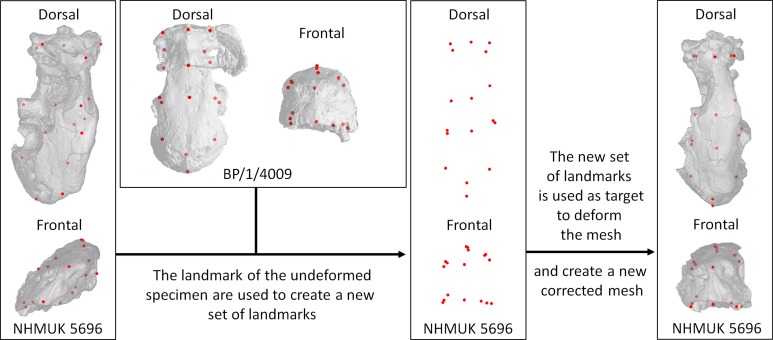
Procedure to correct the diagenetic deformation on NHMUK 5696 using 3D landmarks (red dots).

Retro-deformation: The holotype specimen of *Euchambersi mirabilis* NHMUK R5696 was laterally deformed during fossilization, which strongly affects the shape of the fossae. In order to redescribe the morphology of the fossae and enable accurate reconstruction of the skull of *Euchambersia*, retro-deformation of the specimen was performed (S1 Video) using ISE-MeshTools [37]. We used a protocol of 17 landmarks that were placed on the mesh surface of BP/1/4009, which is not distorted (Fig 3). Then, the same protocol of landmarks was applied to the Mesh surface of the distorted holotype (Fig 3). Finally, we used the landmarks of BP/1/4009 as target landmarks to correct the distortion of the holotype using the “TPS deformation” plugin of ISE-MeshTools (Fig 3). As the holotype is an adult, its proportions are slightly different from those of BP/1/4009 (e.g. the snout is broader and shorter and the sagittal crest is shorter in BP/1/4009; Fig 1A). Thus, the target landmarks could not be scaled up automatically using procrustes superimposition and they had to be manually scaled up to match the size and shape of the holotype. In addition retro-deformation using simple procrust scaling revealed that the diagenetic deformation was not isometric on NHMUK R5696 and thus some target landmarks had to be placed manually (particularly on the left side) in order to account for local heterogeneity (Fig 3). Since this method can correct deformation on a mesh surface only, but not on the μCT slices, the segmentation of the maxillary canal and all the figures related to this structure presented in this paper are not corrected for distortion.

Institutional Abbreviations: BP, Evolutionary Studies Institute (formerly Bernard Price Institute for Palaeontological Research), University of the Witwatersrand, Johannesburg, South Africa; ESRF, European Synchrotron Radiation Facility, Grenoble, France; MS: Faculty of Health Sciences, School of Anatomical Sciences, University of the Witwatersrand, Johannesburg, South Africa; NHMUK, The Natural History Museum, London, United Kingdom.

## Description

### Dentition

**Fig 4:**
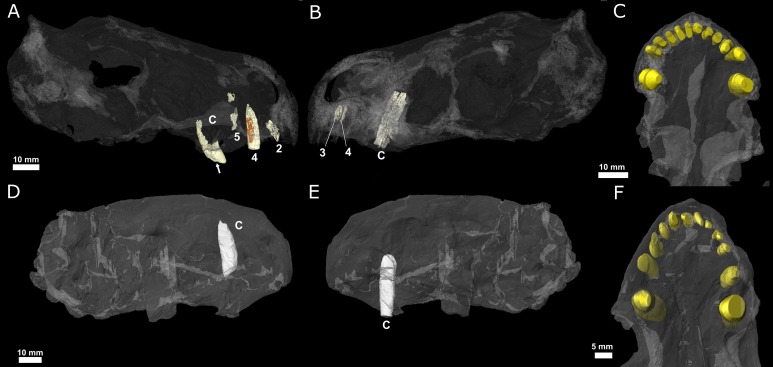
Three-dimensional rendering of the tooth rows of *Euchambersia mirabilis*. A, B, C, BP/1/4009; D, E, F, NHMUK 5696. A, D, lingual face of the left teeth seen through the transparent skull; B, E, lingual view of the right teeth seen through the transparent skull; C, F, ventral view showing the position of the tooth sockets (reconstructed in yellow). The fourth replacement incisor of BP/1/4009 is coloured in orange. Abbreviations: 2, second incisor; 3, third incisor; 4, fourth incisor; 5, fifth incisor; C, canine. The white arrow points to a crack on the right canine of BP/1/4009.

No incisors are preserved in the premaxilla of NHMUK R5696 (Fig 4D and 4E), but the empty alveoli indicate that five teeth would have been present in each premaxilla (Fig 4F). The incisors (I) of BP/1/4009 are incompletely preserved, with root fragments located in the left I2, I4 and I5, and the right I3 and I4 positions (Fig 4). In both NHMUK R5696 and BP/1/4009, the diameter of incisor alveoli increases from the first to the fifth position (Fig 3C and 3F).

**Fig 5:**
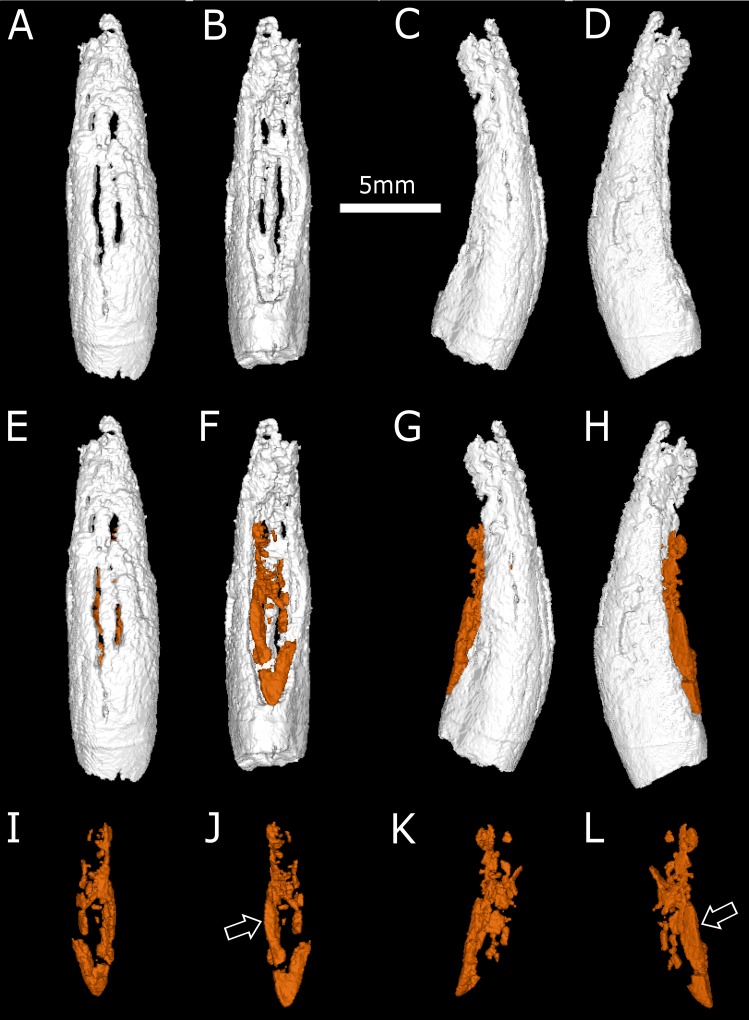
Three-dimensional rendering of the replacement fourth incisor of BP/1/4009. A-D, the fourth incisor alone; E-H, the fourth incisor and the replacement tooth in orange; I-L, the replacement fourth incisor only. A, E, I, labial view; B, F, J, lingual view; C, G, K, mesial view; D, H, L, distal view. The arrows point to the ridge on the replacement tooth.

Prior to μCT-scanning of the specimens, only the left I4 of BP/1/4009 was known [3] and no information on crown morphology of the upper incisors could be gleaned from the preserved fragmentary teeth. A single replacement tooth, preserved in association with the left I4 (Fig 5), offers novel information on incisor crown morphology of *Euchambersia*. The root of the left functional I4 has been etched by the associated replacing tooth, such that resorption of the root took place on the lingual root surface (Fig 5). This is the ancestral condition of tooth replacement present in several amniote lineages [38–47].

Enough of the I4 crown is preserved to determine that, in labial view (Fig 5I), it had the typical theriodont conical appearance. In contrast, the lingual surface of the tooth is slightly concave (Fig 5J–5L). Although it is not well preserved, this replacement fourth incisor seems to have a prominent ridge on its distal edge (Fig 5L). Unlike gorgonopsians and scylacosaurid therocephalians [42, 46, 48–51], the crown of this *Euchambersia* incisor does not bear serrations on either the mesial or distal surfaces (Fig 5I–5L).

**Fig 6:**
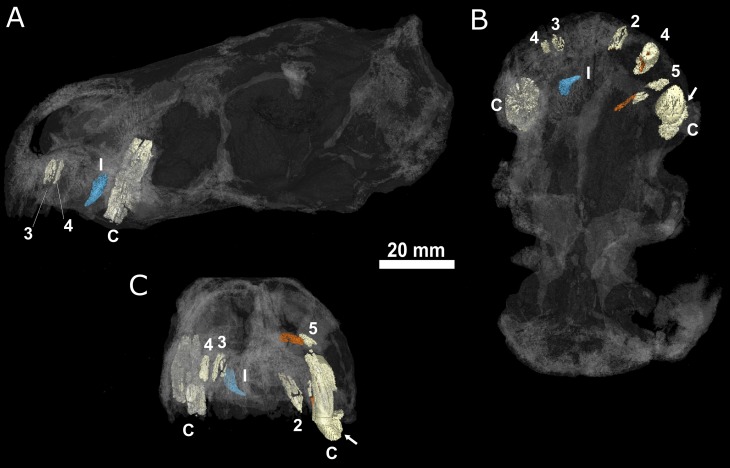
Location of the upper replacement incisor and the *ex situ* lower? incisor of BP/1/4009. A, transparent skull in lateral view showing the labial face of the left hand side; B, teeth and transparent skull in ventral view; C, teeth and transparent skull in rostral view. The fourth replacement incisor is coloured in orange; the *ex situ* incisor is coloured in blue. Abbreviations: 2, second incisor; 3, third incisor; 4, fourth incisor; 5, fifth incisor; C, canine; I, ex situ incisor. The white arrows point to a crack on the right canine of BP/1/4009.

**Fig 7:**
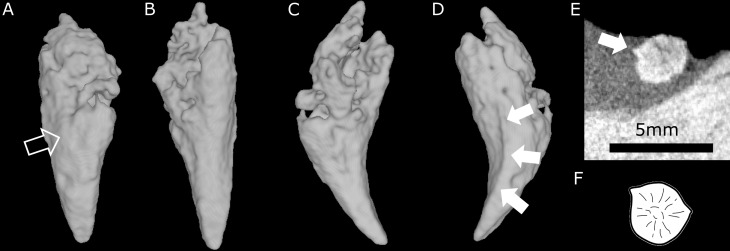
Three-dimensional rendering of the ex situ incisor of BP/1/4009. A, labial view (the empty arrow points to the wear facet); B, lingual view; C, mesial view; D, distal view; E, cross CT section through the middle of the ex situ incisor showing the conspicuous ridge (white arrow); F, cross section of the first right incisor of the lower jaw of an unidentified scylacosaurid therocephalian (= *Glanosuchus*?), redrawn after [51, 52].

In addition to the five incisors preserved *in situ*, an additional tooth is preserved *ex situ*, within the left choana of BP/1/4009 (Fig 6 in blue). The crown of this tooth is more strongly recurved than those preserved *in situ* in the premaxilla. The crown morphology of the isolated *ex situ* tooth also differs from that of the replacement I4. The *ex situ* tooth bears a ridge on its distal face (Fig 7), reminiscent of that figured by van den Heever (ref [51], fig 21, ref [52], fig 27] for the lower dentition of an unidentified scylacosaurid therocephalian (= *Glanosuchus*?) (Fig 7F), and that seen in the ‘incisiform’ teeth of the Nile crocodile (*Crocodylus niloticus*) [53, 54]. A wear facet is present on the labial surface of the *ex situ* crown (Fig 7A), which contrasts with the condition of the superior fourth incisor. Comparison of the tooth morphologies of the *ex-* and *in-situ* crowns differ, providing evidence that the isolated crown may represent a lower incisor and thus, the first and only evidence attributable to a *Euchambersia* lower jaw.

**Fig 8:**
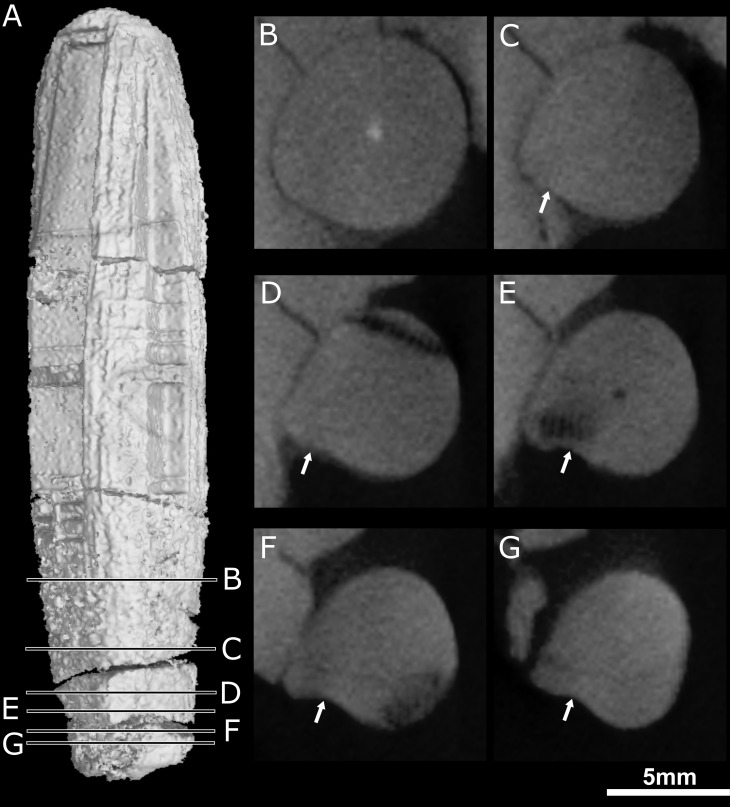
Transverse CT sections through the right canine of NHMUK 5696. The position of each section is shown on the canine (A, the tooth root is up). The white arrows point to the shallow invagination at the base of the ridge.

A cross-section of the right canine crown of NHMUK R5696 shows a ridge on the anterolabial surface of the tooth (Fig 8). A shallow invagination is present on the mesial face of this ridge that widens apically towards the crown apex (Fig 8D–8G). This invagination may represent what has been interpreted as a groove by some authors [11, 13, 27]. The root apex of the left canine is fully-developed as it is completely closed, whereas the root of the right canine is open (Fig 4). The left canine is broken at the neck of the tooth and the root is round in cross-section with no sign of a ridge on the labial surface (Fig 1; S2 Video).

Both maxillary canines of BP/1/4009 are very poorly preserved, and appear to have shattered during preservation, making it difficult to determine the tooth morphology (Figs 4A, 4B and 6; S3 Video). The canine roots are approximately circular in transverse section, and bear no evidence of the anterior groove figured by Brink in 1986 (Fig 6B). The crown of the right canine is not preserved, and that of the left canine is badly weathered and crushed which prevents any interpretation (S3 Video). The mesial surface of the left canine is eroded (Fig 4A), resembling the type of etching that occurs during resorption of a functional tooth by a developing replacement tooth; however, no replacement tooth is preserved in association with the functional canine (Fig 4A). The roots of the canines are somewhat anteriorly projected (Figs 3 and 6), giving the impression that the crowns may have been slightly procumbent. The root of the right canine, which is the best preserved, shows no signs of resorption (Fig 4B). The lack of replacement canines in both specimens of *Euchambersia* is noteworthy. Distichial replacement of the canine teeth, such that the canine locus always bears a functional tooth, is the usual situation in members of the Theriodontia [42, 46]. This lack of replacement canines suggests that either both specimens had reached skeletal maturity, which is unlikely given the size and ossification differences between the two skulls (Figs 1A, 2A and 2B), or that *Euchambersia* may have been more reliant on having both canine teeth present and functional.

*Euchambersia* lacks any postcanine teeth. Above the area corresponding to the postcanine alveolar margin in toothed therocephalians, the maxilla is deeply excavated to form a fossa, which is described below.

### Maxillary fossa and the maxillary canal

The following descriptions are based on the best preserved side of each specimen, i.e. the left for BP/1/4009 and the right for NHMUK R5696. There are some discrepancies in the morphology of the two specimens, especially anatomical details of the maxillary canal. These discrepancies may be attributable to preservation bias, intraspecific variability, or ontogenetic variability.

**Fig 9:**
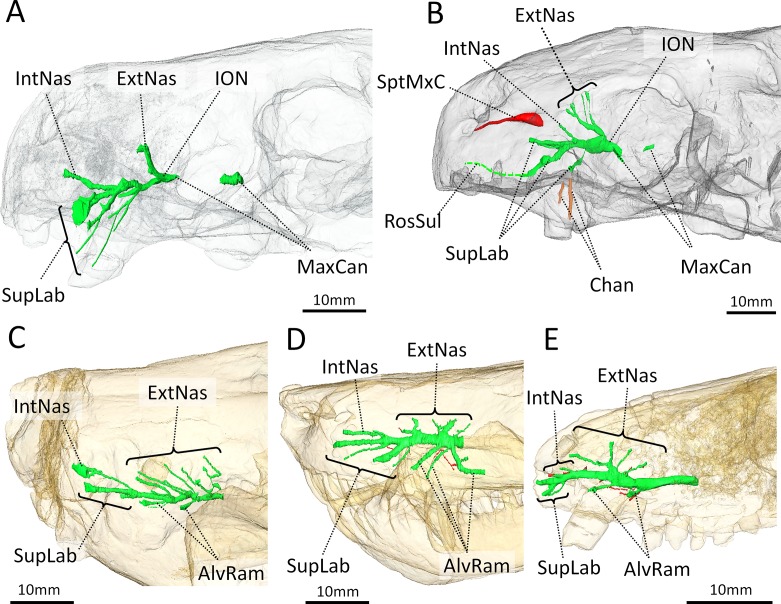
Three-dimensional reconstruction of the maxillary canal in *Euchambersia* and other therapsids in lateral view. A, BP/1/4009; B, NHMUK 5696 (mirrored for comparison); C, *Bauria*; D, *Olivierosuchus*; E, *Thrinaxodon*. Abbreviations: AlvRam, alveolar rami; Chan, channels excavating the maxilla; ExtNas, external nasal rami of the infraorbital nerve; IntNas, internal nasal rami of the infraorbital nerve; ION, infraorbital nerve; MxCan, maxillary canal; RosSul, sulcus on the rostral-most part of the maxilla and premaxilla for a branch innervating the ventral margin of the upper jaw; SupLab, supralabial ramus of the infraorbital nerve; SptMxC, Septomaxillary canal.

The fossa excavates a large surface of the maxilla proximally on the rostrum (average diameter 20mm in BP/1/4009; average diameter 28mm in NHMUK R5696, Table 1) and a part of its posterior margin projects over the orbit (Fig 1A). In BP/1/4009, 38% of the length of the rostrum is occupied by the fossa, and 48% in NHMUK R5696 (Table 1). The fossa is not quite spherical but is instead divided into a shallow dorsal depression, and a deep ventral fossa (Fig 1B). Behind the caudal margin of the canine, a wide sulcus extends along the maxilla from the ventral margin of the fossa to the buccal cavity (Fig 1B and 1C white arrows). Under the assumption that *Euchambersia* was venomous, this sulcus has been interpreted as the notch leading the venom to the buccal cavity [4, 16]. The base of the maxillary fossa is rugose in NHMUK R5696 (it is badly damaged on BP/1/4009) and two foramina, one rostral and one caudal, are present within the fossa (Fig 2C). The μCT images reveal that these foramina lead to the maxillary canal for the trigeminal nerve (Figs 2C and 9), which implies that the maxillary fossa communicates with this bony tube. Hence, the corresponding soft tissues, being the maxillary branch of the trigeminal nerve (CNV_2_), some blood vessels, and maybe a branch of the facial nerve [34], must have passed through the foramina into the fossa and traversed the maxillary fossa in *Euchambersia* (Fig 9). The caudal foramen leads to a posteriorly oriented tube (Figs 2C and 9). This tube disappears caudally inside the endocranial space, 4 and 3 mm posterior to the foramen in BP/1/4009 and NHMUK R5696 respectively. The rostral foramen leads to a tube oriented rostrally. This tube ramifies into the three main branches of the infraorbital nerve (ION) [34] and the point of trifurcation of the three rami is located 6 mm rostral to the foramen in BP/1/4009 (Fig 9A) and 3 mm rostral to the foramen in NHMUK R5696 (Fig 9B). The main stem of the ION and the proximal part of its ramifications are all connected to the canine socket (Fig 2D), which concurs with the role played by the maxillary canal in innervating and carrying nutritive tissues to the canine root [34]. The dorsal-most branch of ION is the external nasal ramus and is oriented dorsorostrally. The external nasal ramus is more ramified in NHMUK R5696 (four rami, Fig 9B) than in BP/1/4009 (one ramus, Fig 9A). In therapsids, the external nasal rami usually ramify into three branches or more [34], as exemplified in *Thrinaxodon*, *Bauria* and *Olivierosuchus* (Fig 9C–9E).

Only one internal nasal ramus is present in *Euchambersia*, as in *Bauria* and *Olivierosuchus* (Fig 9). The ventral-most ramus corresponds to the superior labial rami of the ION (Fig 9). In NHMUK R5696, the middle branch of the superior labial ramus opens rostrally to a groove that runs parallel to the margin of the buccal cavity (Fig 9B). This branch of the ION and the accompanying vessels may have innervated and supplied the ventral margin of the upper jaw. In BP/1/4009, the two ventral-most branches emit thin ramifications oriented toward the surface of the bone next to the position of the canine (Fig 9A). No such ramifications are present in NHMUK 5696, but some dorso-ventrally oriented sulci on the surface of the maxilla seem to prolongate the superior labial rami (Fig 9B, in orange). Finally, the complete absence of alveolar rami of the maxillary canal in both NHMUK R5696 and BP/1/4009, compared to other therapsids, is noteworthy (Fig 9) [34]. This absence can be attributed to the absence of post-canine dentition in *Euchambersia*. As such, the maxillary canal is limited to the section carrying the ION in this taxon.

## Discussion

### The dentition of *Euchambersia*, ridged or grooved?

**Fig 10:**
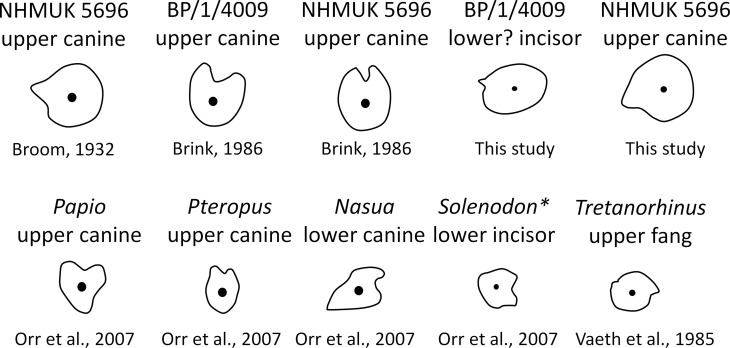
Cross sections through the teeth of various mammals redrawn from literature and compared to *Euchambersia*. Mesial is on the top, labial is on the right. * indicates venomous species.

**Fig 11:**
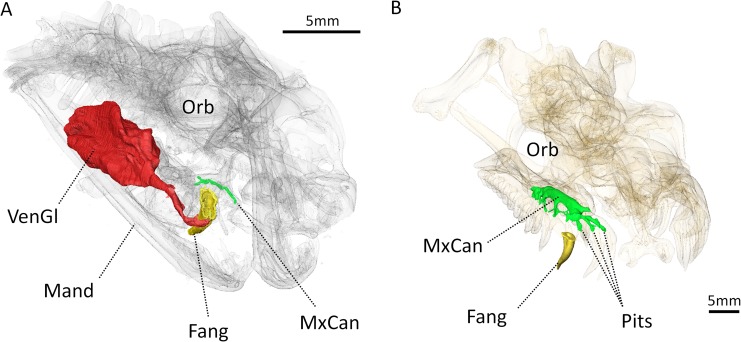
**Three-dimensional rendering of the transparent skulls of (A) a rinkhals (*Hemachatus haemachatus*) and (B) a python (*Python* sp.).** Oblique view showing the relationship of the mummyfied venom gland (in red), with the fang (in yellow) and the maxillary canal (in green). Abbreviations: Fang, functional fang; Mand, mandible; MxCan, maxillary canal; Orb, orbit; Pits, foramina for the pit organ; VenGl, venom gland.

**Fig 12:**
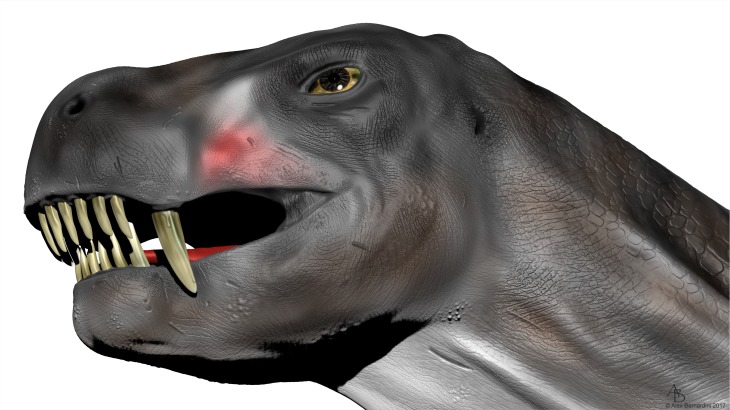
Reconstruction of *Euchambersia*. Oblique ventro-lateral view showing the ridged dentition. Artwork by Alex Bernardini (Simplex Paleo 2017).

Brink [27] erroneously figured a deep groove on the anterior margin of the canine which is evocative of the opistoglyphous maxillary teeth of a venomous snake (Figs 10 and 11A); however, no such groove is present on the canine (*contra* [11, 13, 27]). Only a shallow invagination is present at the base of the canine ridge in cross section (Fig 8). Even if not deeply grooved, the teeth of *Euchambersia* were at least ridged (Figs 7 and 8). Micro-CT scan of the canine confirms the presence of a canine ridge on only the crown (not the root) of NHMUK R5696 (Fig 8), as suggested by Broom [29, 30]. The crowns of the canines of BP/1/4009 are not preserved and the root bears no continuity of a ridge. Unfortunately, no replacement canine is preserved to aid interpretation (Fig 4). The presence of a labial ridge on the canine was documented in the original description by Broom [29, 30]. Our μCT observations confirm the presence of this ridge on the canine of the holotype (Fig 8) and add the occurrence of a ridged (lower?) incisor in BP/1/4009, the only tooth with a well-preserved crown in any specimen of *Euchambersia* (Fig 7). Another erupting *in situ* upper incisor may also have been ridged, but its morphology is less clear (Fig 5). This evidence strongly suggests that: (i) *Euchambersia* may have had recurved (lower?) incisors; (ii) ridged dentition characterizes *Euchambersia*; and (iii) the presence of a ridge was not limited to the canine but was present on all the teeth (as there are no post-canines, the dentition comprises only incisors and canines) (Fig 12).

### The venom gland hypothesis into question

With its broad and robust snout, characteristically marked by two large and deep lateral maxillary fossae, *Euchambersia* presents a unique morphology among extant and extinct vertebrates. A similar condition is present in the skull of some extant ruminants, particularly the Muntjac deer, which has a pre-orbital fossa filled with a sac for secretions emitted from the sweat and sebaceous glands that are used mainly for scent marking [55, 56]. A variety of mammals, reptiles and birds also have enlarged preorbital glands of various functions (e.g. salt gland, lacrimal gland) which naturally supported the conclusion that the maxillary fossa of *Euchambersia* housed a gland of some sort [57, 58]. Broom [29, 30] argued for a parotid (salivary) gland, but Boonstra [7] and Lehman [9] opposed this idea, pointing out that the parotid gland is always located post-orbitally. Boonstra [7] argued that the maxillary fossa of *Euchambersia* housed a modified lacrimal or labial gland, whereas Lehmann [9] proposed that it was a modified Harderian gland. Lehmann’s 1961 hypothesis is less likely since Harderian glands are located inside the orbit [59]. Grine et al. [57] argued that the pre-orbital fossa of the cynodont *Diademodon* could have housed a salt-gland, an interpretation based on the presence of sulci that ramify rostrally from the fossa on the surface of the snout in some specimens of *Diademodon* [57]. *Euchambersia* displays no evidence of such sulci and its fossa is located more ventrally than that of *Diademodon*, suggesting that the two fossae may not be homologous.

Nopcsa [4] pointed out that Broom failed to recognize the actual function of the huge maxillary fossa in *Euchambersia* because he did not correlate it to the peculiar shape of the canine which bears a sharp ridge on its labial face. Such a ridge could have facilitated the injection of a substance secreted by an organ located inside the fossa, and therefore suggests that the maxillary fossa was for a venom gland [4]. Therefore, as far as it can be traced in the literature, the huge maxillary fossa has always been interpreted as the place for a secretive gland (venom or saliva) [4, 26, 29, 30].

As argued by [4], venom would have been injected to the prey through the bite after the venom had passively impregnated the saliva through the deep notch located posterior to the canine (Fig 1, white arrow). Here we point out an additional possible pathway for the secretions of the venom glands. In both NHMUK R5696 and BP/1/4009, the base of the maxillary fossa is pierced by two foramina. The μCT scan based analyses reveal that these foramina lead to osseous tubes that run inside the maxillary canal (Fig 9). In *Euchambersia*, this bony canal thus extends through the maxillary fossa (Fig 2C) and then, as in other therapsids [34, 60], it merges with the canine tooth socket for a distance before exiting the skull through the foramina on the rostrum (Fig 9). In extant tetrapod species the maxillary canal chiefly carries the maxillary division of the trigeminal nerve (CNV_2_), but it also provides a passage for blood vessels (infraorbital arteries and veins) and a branch of the facial nerve [61–63]. Thus, it is as likely that the maxillary canal may have also carried venom directly to the canine allowing for a more active injection mechanism. Additional sulci on the surface of the maxilla located directly above the canine could also have participated in the process (in orange on Fig 9).

**Fig 13:**
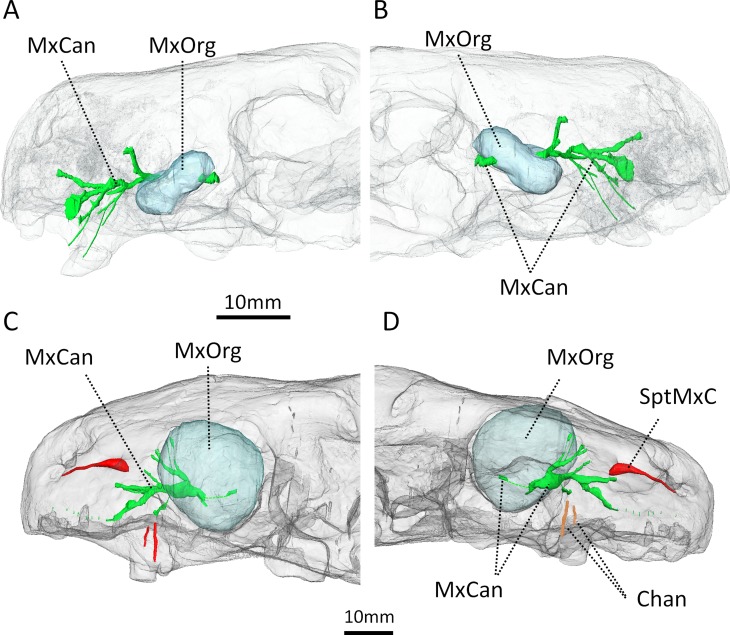
Three-dimensional rendering of the hypothetical organ that filled the maxillary fossa and its relationship with the maxillary canal (in green). A, B, BP/1/4009; C, D, NHMUK 5696 (mirrored for comparison). A, C, lateral view; B, D, medial view. Abbreviations: Chan, channels excavating the maxilla; MxCan, maxillary canal; MxOrg, maxillary organ; SptMxC, septomaxillary canal.

In conclusion, venom is a secretion produced in a specialised gland in one animal, and delivered to a target animal through the infliction of a wound [6, 64, 65]. Following the definition of Bücherl [66], a venomous animal must possess: (i) at least one venom gland; (ii) a mechanism to deliver the venom; and (iii) an apparatus with which to inflict a wound for venom delivery. *Euchambersia* has shown potential evidence for all of these attributes: (i) the large maxillary fossa which may have housed a toxin producing gland (Figs 1A and 13); (ii) a large sulcus leading to the mouth cavity (Fig 1B and 1C), or the maxillary canal leading to the base of the canine (Figs 9 and 13) for transport of the venom; and (iii) ornamented teeth for wounding and delivering venom to the target prey animal (Figs 7 and 8). Thus, the cranial morphology of *Euchambersia*, as revealed by this study, fulfills these criteria and supports the venomous *Euchambersia* hypothesis, though it does not definitively prove it.

With the exception of snakes, the venom gland is usually associated with the lower jaw in extant venomous vertebrates such as in the Caribbean *Solenodon*, the Komodo dragon or the Gila Monster [6, 16, 23]. In venomous snakes, in which the venom gland is located post-orbitally within the upper jaw, the venom does not flow through the maxillary canal but is directly carried to the grooved-teeth by an independent duct (Fig 11A) [64, 65]. The absence of an extant analogous condition to that observed in *Euchambersia* makes comparisons impossible and the validity of the venomous hypothesis difficult to assess. Alternatively, if a venom gland was absent in *Euchambersia*, the infraorbital artery and veins could have supplied nutrients and drained waste from the extensive organ filling the maxillary fossa, regardless of its nature (Fig 13).

As stated above, many non-venomous species do possess grooved or ridged teeth (Fig 10). Among non-venomous snakes, prominent tooth ribbing seems to be correlated with slippery and mucus-covered prey hunting (fishes and invertebrates) because a ridge would reduce suctional drag during tooth disengagement [31]. In addition, among mammals, a grooved canine may play a role in grooming or sharpening the teeth [16, 67] and a ridged or sharp canine is accompanied with a preorbital depression or fossa in e.g. baboons and muntiacs, which weakens the argument for envenoming capacity [16, 32]. The presence of ridged tooth morphology and its association with the maxillary fossa thus only supports, but does not indicate without doubt that *Euchambersia* was venomous. The shallow invagination and the ridge on the canine of *Euchambersia* do not appear to be the result of the presence of a wear facet, which at least, would exclude a role in sharpening the teeth.

### A neural or sensory element as an alternative hypothesis

As described and discussed above, the maxillary canal opens into the maxillary fossa (Fig 2C), so whatever was passing through this canal (CNV_2_, facial nerve and/or blood vessels), the maxillary fossa had a direct relationship with these soft tissue structures, and particularly the somatosensory system. Indeed, the morphology of the maxillary canal has been directly related to the evolution of facial sensitivity and motility in therapsids [34, 68]. The CNV_2_, which runs through that canal, is the nerve responsible for facial sensitivity and the accompanying vessels innervate and supply the skin and facial muscles in amniotes [61, 69, 70]. In reptiles, the CNV_2_ is the major nerve inside the canal, but it may be accompanied by a diminutive branch of the facial nerve [61, 69].

In *Euchambersia*, there is evidence for the presence of only the inferior orbital nerve (ION), which is the main branch of the CNV_2_. The morphology of the ION differs significantly between *Euchambersia* and other therocephalians (Fig 9) [34], and the maxillary canal is exposed caudally inside the maxillary fossa (Figs 9 and 13) which is a unique condition among therapsids [34]. This last condition implies that a segment of the CNV_2_ extended outside the skull at the level of the fossa (Fig 13). Therefore *Euchambersia* is unique in the morphology of its maxillary canal and the connection of this canal with the maxillary fossa suggests that the main tissue occupying this structure (i.e. the CNV_2_) was very specialized in this taxon. This leads us to consider the possibility that in *Euchambersia* this fossa housed a specialized sensory organ analogous to the thermosensitive pit organ of pit vipers, and some boid and python snakes [71] (Fig 11B) or a nervous ganglion (Fig 13).

In this respect, it is noteworthy that *Euchambersia* is among the few therocephalians of its time to lack a parietal foramen, the external opening for another important sensory organ, the pineal eye [72]. In extant species in which it is present (the tuatara and some lizards), the pineal eye is involved in the monitoring of different life cycles such as reproduction cycles and regulation of time exposure to the sun and body temperature in accordance with days, nights, and seasons [73]. In most land vertebrates, the lateral eyes compensate for the absence of the extra third eye, but in pit vipers, experiments have shown that it is the facial pit organ that helps to make the correct thermoregulatory decision [74]. Building on the hypothesis that the facial organ of *Euchambersia* was a sensory organ, and given the absence of a parietal foramen, this hypothetical organ may have compensated for the absence of a pineal eye in *Euchambersia*. However, this would be a completely unique condition among tetrapods as no extant species is know to display such a dramatically hypertrophied facial organ. Moreover, many therapsid species lacking any trace of a preorbital fossa are also devoid of a parietal foramen [72]. Finally, as argued above, the need for blood supply of a venom gland (or any other organ whatever its nature), the absence of post-canine teeth, and the deformation induced by the presence of the fossa could also account for *Euchambersia*’s unique maxillary canal features. Also, the sensory organ hypothesis is less satisfying because it does not account for all the anatomical specializations of *Euchambersia* (e.g. the ridged dentition), contrary to the venom gland hypothesis.

## Conclusion

Because of the uniqueness of its skull anatomy, *Euchambersia mirabilis* is and will remain a puzzling species, particularly in regard to its palaeobiology. Our reappraisal shows that despite some reservations, the venom secreting gland hypothesis remains the most plausible explanation to account for the very peculiar morphology of the maxilla and dentition of *Euchambersia*. The discovery of an *ex situ* tooth in the choana of the newest specimen (BP/1/4009) documents, for the first time, the crown morphology of the species and confirms that the teeth of *Euchambersia* were ridged along their length. The *ex situ* tooth is the first recorded evidence of the lower jaw anatomy of *Euchambersia* and in the near future, may help to identify and refer additional mandibular material to this extremely derived and intriguing taxon.

## Supporting information

S1 Video Video showing the effect of the correction of diagenetic deformation on NHMUK 5696 in various views. (AVI)

S2 Video Video of CT axial sections through the snout of NHMUK 5696. Scale bar: 10mm.
(AVI)

S3 Video Video of CT axial sections through the snout of BP/1/4009. Scale bar: 15mm.
(AVI)
